# Study of Montmorillonite Clay for the Removal of Copper (II) by Adsorption: Full Factorial Design Approach and Cascade Forward Neural Network

**DOI:** 10.1155/2013/342628

**Published:** 2013-12-18

**Authors:** Nurdan Gamze Turan, Okan Ozgonenel

**Affiliations:** ^1^Department of Environmental Engineering, Engineering Faculty, Ondokuz Mayis University, 55139 Kurupelit, Samsun, Turkey; ^2^Department of Electric and Electronic Engineering, Engineering Faculty, Ondokuz Mayis University, 55139 Kurupelit, Samsun, Turkey

## Abstract

An intensive study has been made of the removal efficiency of Cu(II) from industrial leachate by biosorption of montmorillonite. A 2^4^ factorial design and cascade forward neural network (CFNN) were used to display the significant levels of the analyzed factors on the removal efficiency. The obtained model based on 2^4^ factorial design was statistically tested using the well-known methods. The statistical analysis proves that the main effects of analyzed parameters were significant by an obtained linear model within a 95% confidence interval. The proposed CFNN model requires less experimental data and minimum calculations. Moreover, it is found to be cost-effective due to inherent advantages of its network structure. Optimization of the levels of the analyzed factors was achieved by minimizing adsorbent dosage and contact time, which were costly, and maximizing Cu(II) removal efficiency. The suggested optimum conditions are initial pH at 6, adsorbent dosage at 10 mg/L, and contact time at 10 min using raw montmorillonite with the Cu(II) removal of 80.7%. At the optimum values, removal efficiency was increased to 88.91% if the modified montmorillonite was used.

## 1. Introduction

The high degree of industrialization worldwide has resulted in environmental problems [1, 2]. Greater environmental awareness in both the public and regulatory spheres in recent years has necessitated greater treatment of industrial effluent [3]. Unproductive ways to reduce heavy metal ions in wastewater may bring long-term risks to the ecosystem and humans. Harmful toxic heavy metals that are discharged by several industries include cadmium, mercury, lead, chromium, copper, nickel, and zinc [4].

Copper is one of the most important metals. It is frequently found in effluents discharged from industries such as mine drainage, galvanizing plants, natural ores, and municipal wastewater treatment plants. Copper is not biodegradable and travels through the food chain via bioaccumulation. The increase of Cu(II) in human body causes some major diseases such as brain, skin, pancreas, and heart diseases [5].

Some treatment technologies such as precipitation, ionic exchange, and adsorption have been applied for heavy metal removal in aqueous solution. Among them, adsorption receives considerable interest in heavy metal removal due to its high efficiency, easy handling, cost effectiveness, and the availability of different adsorptive materials [6–11]. Although many adsorbents are used in adsorption studies, activated carbon is mainly used in wastewater treatment all over the world. However, it is not cost-effective procedure and needs chelating agents to enhance its performance [12]. Recently, adsorption studies have been paid attention on using low-cost, effective sorbents for heavy metal removal, and the sorption behavior of several natural materials and waste products has been examined [13–16].

Clay is a potentially good adsorptive material because of its large surface area, high cation exchange capacity, chemical and mechanical stability, and layered structure [17]. The adsorption of heavy metal into natural clays has recently been studied by various researchers. Among natural clays, montmorillonite acts as a potential ionic exchanger for heavy metals due to its low-cost, high abundance, easy manipulation, and harmlessness to the environment [18–24]. Therefore, clays have been used in many studies, mainly montmorillonite, to show their effectiveness for removal of metal ions from aqueous solutions. However, statistical and optimization studies on heavy metal removal using montmorillonite under various physicochemical parameters are restricted and very rare.

In this work, 2^4^ full factorial design and cascade forward neural network (CFNN) were used to estimate the removal of Cu(II) from industrial leachate by adsorption process. Of the two approaches, CFNN was first applied to adsorption studies. The inputs include initial pH, adsorbent dosage (mg/L), contact time (min), and adsorbent type (raw or modified montmorillonite). The sorption of Cu(II) concentration is considered as output. 2^4^ full factorial design requires 16 experiments followed by ANOVA, *F*-test, and residue analysis to model the batch experimental system. However, CFNN does not require extensive experimental studies due to its structural advantages and only 8 tests need to train the network. Therefore, CFNN represents a powerful tool for the classification of the relevant parameters and their interactions. Furthermore, it was found to be more economical and easier to implement.

## 2. Materials and Method

### 2.1. Materials

#### 2.1.1. Montmorillonite

**Figure 1:**
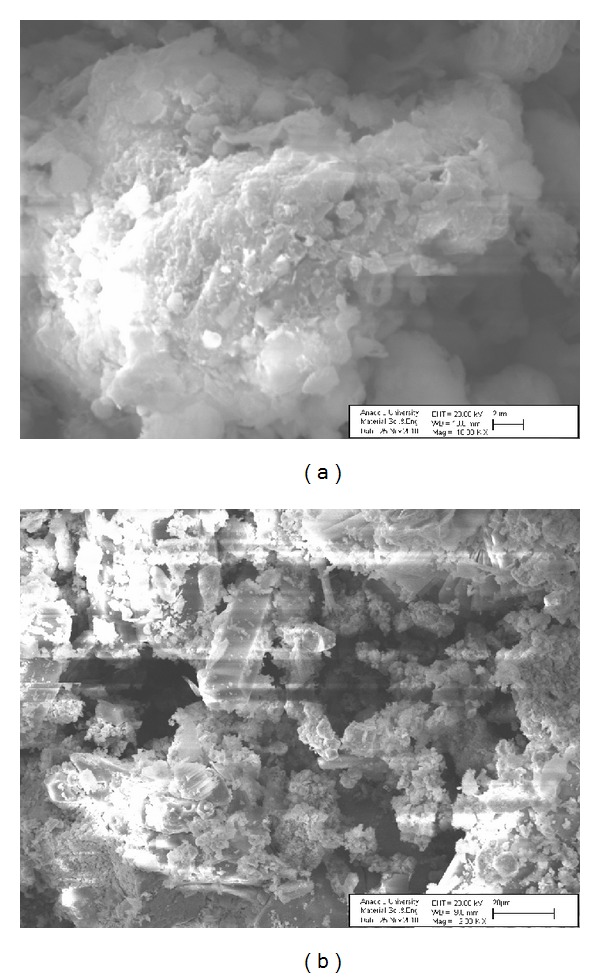
SEM micrographs of the montmorillonite (a) and industrial waste (b) samples.

Samples of montmorillonite were obtained from the Bensan Activated Bentonite Company. For experimental studies, the mineral was washed with distilled water to remove any nonadhesive impurities and small particles and then dried at 70°C for 24 h to remove moisture. The samples were sieved through a 0.6 mm sieve and used without any treatments. Finally, obtained activated sawdust granules were stored in separate vacuum desiccators for further use. The chemical composition of the montmorillonite used in this work is given in Table 1. As seen in Table 1, montmorillonite contains significant levels of SiO_2_ (64.11%) and Al_2_O_3_ (18.04%), while the contents of other metal oxides are less than 10%. Scanning electron microscopy (SEM) technique was used to monitor the surface physical morphology of the waste and montmorillonite.  Figure 1 shows the SEM photograph of the montmorillonite.

#### 2.1.2. Industrial Waste

The copper flotation waste was obtained from the Elektrosan Elektrocopper Industry (Samsun, Turkey). The chemical composition of the waste was given in Table 1.

### 2.2. Leaching Procedure

The toxicity characteristic leaching procedure (TCLP), as given in the EPA's SW846, was applied for analyzing pollution potentials of the industrial waste. The TCLP consists of extracting contaminants from a waste with an appropriate extraction fluid. The extraction fluid depends on the alkalinity of the waste. After extraction, metal concentration is analyzed [25, 26].

### 2.3. Batch Mode Adsorption Studies

The batch studies were performed to study the removal of Cu(II) from aqueous leachate of industrial waste. A leaching solution containing 1 g of adsorbent was mixed by stirring the mixture at 200 rpm with a 100 mL aqueous leachate of waste in a flask placed in a shaking incubator, keeping constant working temperature. An aliquot of the solution was withdrawn at predetermined time intervals and was filtered to remove adsorbent particles. The Cu(II) concentration in the filtrate was subsequently determined using a UNICAM 929 Model Atomic Absorption Spectrophotometer. The adsorption tests were continued until the equilibrium concentration was reached. The effects of initial pH, adsorbent dosage, and contact time on the amount of Cu(II) adsorbed were examined.

The parameters,* sorption capacity *of the substrateand* sorption efficiency* of the system, were used to test the system at equilibrium. *Sorption capacity* of the substrate (*q*_*e*_) is expressed in terms of metal amount sorbed on the unitary natural sorbent mass (mg g^−1^), and *sorption efficiency* of the system (*R*_em_%) is indicated from the percentage of removed metal ions relative to the initial amount. These parameters have been calculated as in (1).

(1) $$\begin{array}{c} q_{e}=\frac{C_{i}-C_{e}}{W}V,\\ R_{\text{em}}\%=\frac{C_{i}-C_{e}}{C_{i}}\times 100, \end{array}$$

where *C*_*i*_ is the initial concentration of metal ions in solution (mg L^−1^), *C*_*e*_ is the final concentration of metal ions in solution (mg L^−1^), *V* is the volume of the solution (*L*), and *W* is mass of adsorbate (*g*).

All experiments were duplicated to increase the reliability of the experimental procedure, and the average values of *R*_em_% were taken into account.

### 2.4. Apparatus

Adsorption studies were done by a shaking incubator (Stuart SI500). Heavy metal concentration in the leaching solution was carried out by an Atomic Absorption Spectrophotometer (UNICAM 929 Model). The chemical compositions of the materials were evaluated by XRF using an X-ray fluorescence spectrophotometer (RIGAKU Model). The powdered samples were sieved under 63 *μ*m for homogeneous particle size. The samples were mixed with lithium borate flux, heated in a platinum crucible to between 900 and 1300°C, and then cast in a dish to produce a homogeneous glass-like bead. The beads were placed in XRF to obtain the experimental analysis of the samples. Microstructural investigations of the materials were analyzed by using a scanning electron microscopy (Zeiss EVO 50EP). The powdered samples were attached to the stage surface using carbon tape. All samples were coated with Au-Pd target metal under argon atmosphere by sputter coater. The coated samples were placed in SEM chamber at high vacuum. The surface micromorphology of materials was investigated. The solution pH was carefully adjusted using a pH meter (Mettler Toledo MP220) [27].

### 2.5. Statistical Approaches

Classical methods, such as one-factor-at-a-time experiments, do not characterize the combined effect of all the factors involved [28, 29]. This method is, of course, time-consuming and requires many experiments to identify optimum levels. In this study, the combined effect of initial pH, adsorbent dosage, contact time, and type of adsorbent (raw and modified montmorillonite) has been investigated by statistical experimental design such as 2^4^ full-factor experimental design (FFED).

FFED is based on mathematical and statistical techniques to evaluate the relative significance of numerous effecting factors even in the existence of complex interactions [30–32]. Design experiments, regression analysis through ANOVA and *F*-test, and optimization are the steps of FFED. The most important aim of FFED is to identify the optimum levels of the analyzed factors [33, 34]. The application of FFED in adsorption studies results in improved product efficiency, reduced process versatility, closer verification of the output reaction to nominal and objective necessities, and shortened development time and overall costs [35, 36]. This method is broadly used in chemical engineering, in particular to optimize the adsorption process.

After FFED is conducted, the response variable can be represented by linear, interaction, quadratic, and pure quadratic. Regression equations, *R*, can be described as in

(2) $$\begin{array}{c} R_{\text{linear}}=X_{0}+X_{1}A+X_{2}B+X_{3}C+X_{4}D, \end{array}$$

(3) Rinteraction=X0+X1A+X2B+X3C+X4D +X12AB+X13AC+X14AD +X23BC+X24BD+X34CD,

(4) Rquadratic=X0+X1A+X2B+X3C+X4D+X12AB +X13AC+X14AD+X23BC+X24BD+X34CD +X11A2+X22B2+X33C2+X44D2,

(5) Rpure-quadratic=X0+X1A+X2B+X3C+X4D+X11A2 +X22B2+X33C2+X44D2,

where *X*_*s*_ represent constant, linear, interaction, and quadratic coefficients, while *A*, *B*, *C*, and *D* represent initial pH, adsorbent dosage (mg/L), contact time (min), and adsorbent type (raw and modified montmorillonite), respectively.

Four factors were studied for Cu(II) removal by montmorillonite; their low and high levels are given in Table 2.

A total of 16 experiments were conducted according to the design in Table 3. Mean values were used, and maximum deviation was found to be  ±2.85%.

The outputs were examined using Minitab Software, and the main and interaction effects were identified. The main effects of related factors are the change in the *R*_em_% linked to the levels of the analyzed factor. The effects, regression coefficients, and the related probability (*P*)  values are given in Table 4. According to the *P* values, the batch experimental system is represented by a regression analysis as in (2) to (5).

According to Table 4, main effects and *B*∗*D* interaction effect are significant since *P* value is less than 0.05 (confidence of interval). Analysis of variance (ANOVA) test also proves that main effects are mostly significant. Identification of the major main and interaction effects of the factors affecting *R*_em_% was achieved by ANOVA (Table 5) [37–39].

In Table 4, *DF* is the degrees of freedom, *SS* is the sum of squares, *MS* is the mean squares, *F* value is calculated by dividing the factor *MS* by the error *MS*, and probability (*P*) value is used to verify whether a factor is significant.

The simplified regression equation for Cu(II) removal efficiency by montmorillonite is in

(6) $$\begin{array}{c} R_{\text{em}}\%=80.11+8.85A+5.36B+1.5C+4.38D+0.51BD. \end{array}$$

Since *P* value of *BD* interaction effect (0.048) is almost 0.05, this effect can be excluded from the regression equation. Then (5) simplifies to (7). Note that all main and interaction effects have positive influence on removal efficiency. Consider

(7) $$\begin{array}{c} R_{\text{em}}\%=80.11+8.85A+5.36B+1.5C+4.38D. \end{array}$$

**Figure 2:**
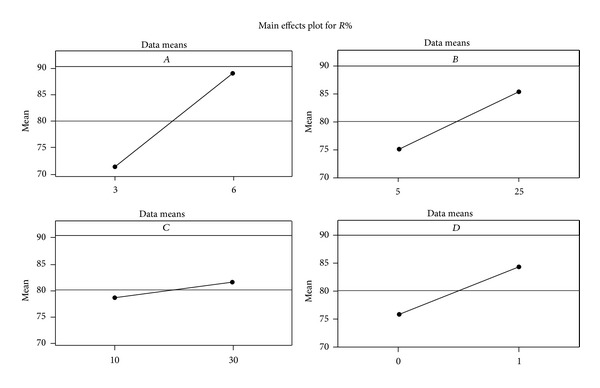
Main effects plot for removal efficiency.

The main effects (*A*, *B*, *C*, and *D*) symbolize deviations of the average *R*_em_% between the selected levels for each one (Figure 2).

Upon changing the levels of *A*, *B*, *C*, and *D* factors from min to max, the *R*_em_% is amplified by 19.89%, 12.54%, 3.67%, and 10.39%, respectively. Consequently, their effects are considered positive.

**Figure 3:**
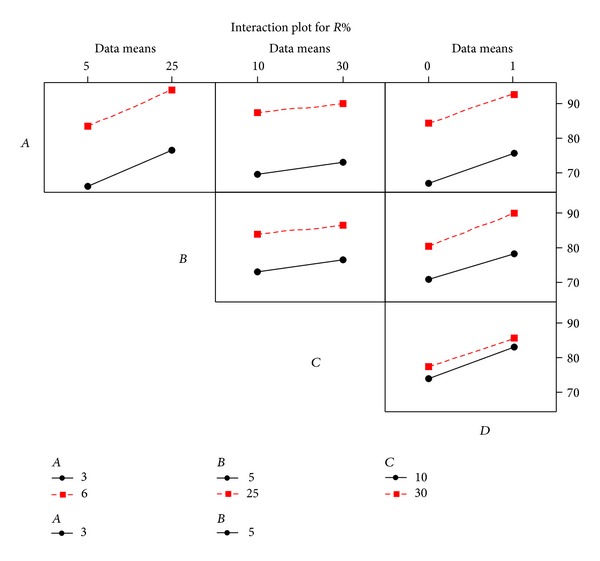
Interaction effects plot for removal efficiency.

The interaction effects plots present the mean response of two factors at all feasible combinations of their settings (Figure 3).

**Figure 4:**
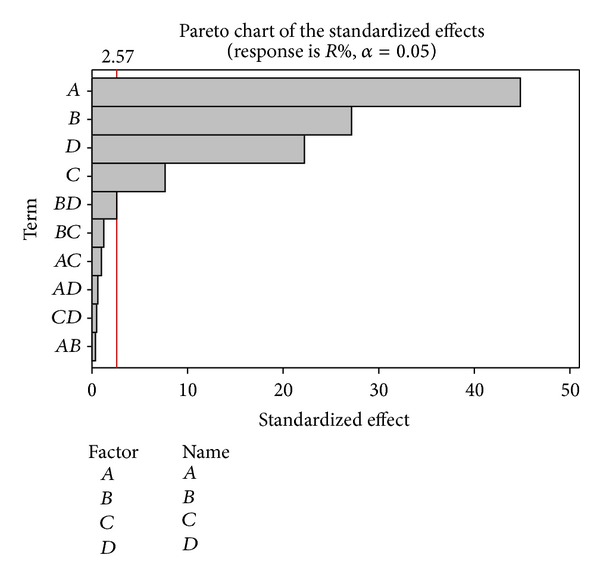
Pareto chart of standardized effects on removal efficiency.

The interaction plots demonstrate that only *B* and *D* interaction effects are almost as significant with a value of 0.048, which is regarded as a weak interaction effect. Removal efficiency is increased by 9.85% if *B* is in low value and *D* is changed from 0 to 1. Similarly, removal efficiency is increased by 10.84% if *B* is in high value and *D* is changed from 0 to 1. Student's *t*-test was used to see if calculated effects were notably different from zero. For a 95% confidence interval and 10 degrees of freedom, the *t*-value is equal to 2.57.  Figure 4 presents this estimation as a Pareto chart. The vertical line indicates minimum statistically significant effect magnitude. Values shown in the horizontal columns are Student's *t*-test values of each effect.

Column values are calculated as 44.90 for *A*, 27.2 for *B*, 22.26 for *D*, and 2.6 for *C*. Based on *F*-value and Student's *t*-test value, the interaction effects can be eliminated, and 8 can describe the batch experimental system by montmorillonite.

It is noteworthy that (7) is based on some assumptions (Table 2) [40, 41]. Therefore, residual plots must be analyzed to make sure of the model adequacy and check whether this hypothesis of regression has been fulfilled.

**Figure 5:**
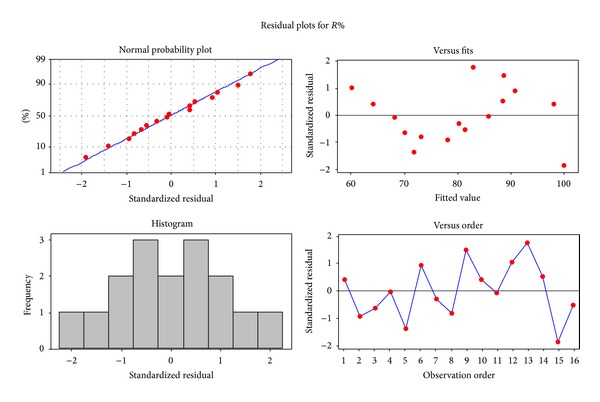
Residual plots for removal efficiency.

The residuals are the differences between experimental and predicted values of *R*_em_%.  Figure 5 shows the normal probability plot, variation of the fitted values, and histogram of the residuals for Cu(II) removal system.

It is obvious that experimental runs present a normal distribution. The residuals are not linked to the estimated response. This is easily checked by plotting the residuals versus estimated values (Figure 5). Note that no pattern should be seen in residual plot (Figure 5).

The histogram distribution of residual *R*_em_% for Cu(II) indicated that the data points larger than 2 are considered an outlier (Figure 5). The corresponding data points, which have values larger than 2, are between −0.75 to −0.25 and 0.25 to 0.75 in histogram plot (Figure 5).

**Figure 6:**
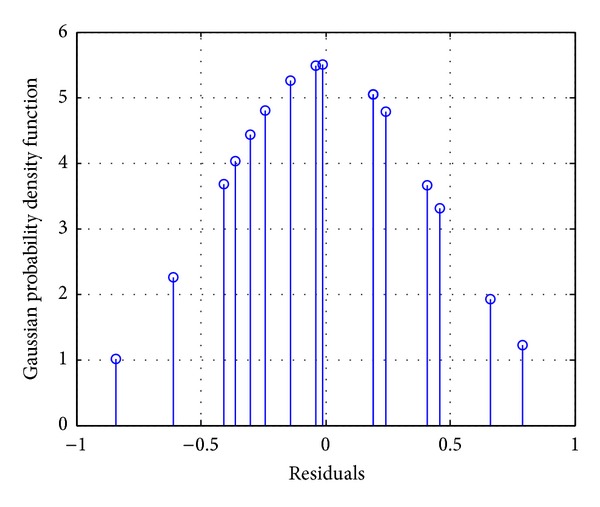
Gaussian probability density function of the residuals.

Gaussian probability density function was applied to the residuals to check the normality and prove the above statement.  Figure 6 shows the Gaussian probability density function of the residuals.

Two important criteria also calculated the probability density function. The skewness value was found to be −0.57, which means that the value is between ∓1.96 (for 95% confidence interval), and the curve presents a Gaussian shape. Moreover, the kurtosis value was found to be 1.97, which means that the value is almost near to 3, presenting a Gaussian shape.

**Figure 7:**
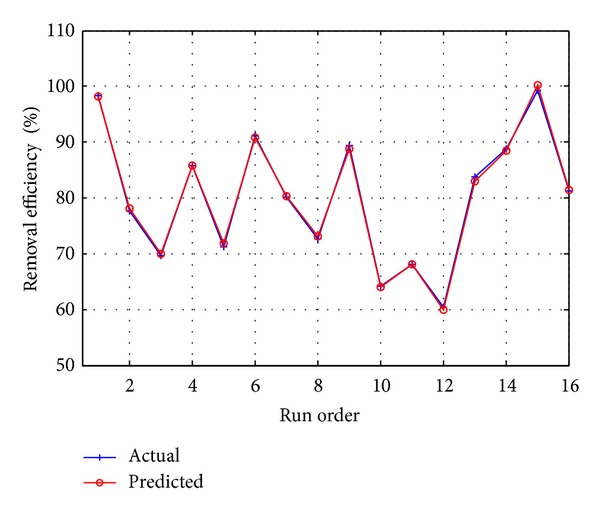
Actual and predicted Cu(II) removal efficiencies.

Figure 7 shows that the estimated values of *R*_em_% obtained from the regression equation and the actual *R*_em_% were almost acceptable with adjusted *R* ^2^ = 99.55% value.

Optimizing the operational conditions is an important step for batch experimental studies. Different combinations of the analyzed factors were tried to maximize the removal efficiency. Both adsorbent dosage and contact time options were costly, so these factors were chosen as minimums in optimization steps. Optimization begins at a random starting point with the weight and importance values of 1, and different combinations were tried to maximize the output response.

Weight number is used for shaping of the desirability function, while importance value is used for specifying the comparative importance of the response. The lower removal efficiency was chosen at between 80% and 99% during the optimization process.  Table 6 gives the local solutions and predicted Cu(II) removal efficiency. Adsorbent type (*D*) is switched from 0 to 1 during optimization process.

**Figure 8:**
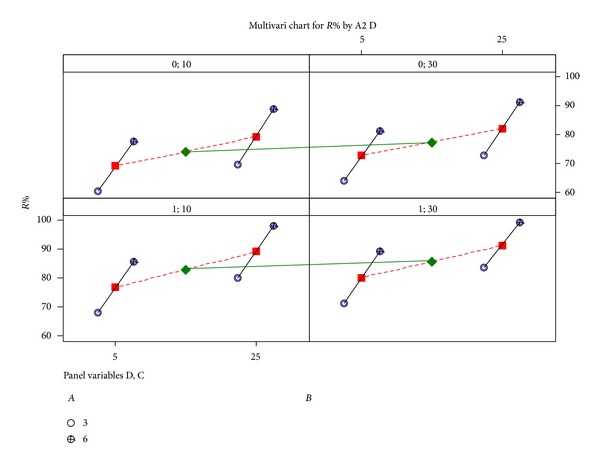
Multivari chart for analyzed factors.

Since it is preferable to work at the lowest adsorbent dosage, contact time, and unmodified material type due to economic aspects, the fourth local solution can be acceptable (Table 6). A multivari chart also proves the results of optimization solution on Cu(II) removal (Figure 8), and optimum levels can easily be selected.

**Figure 9:**
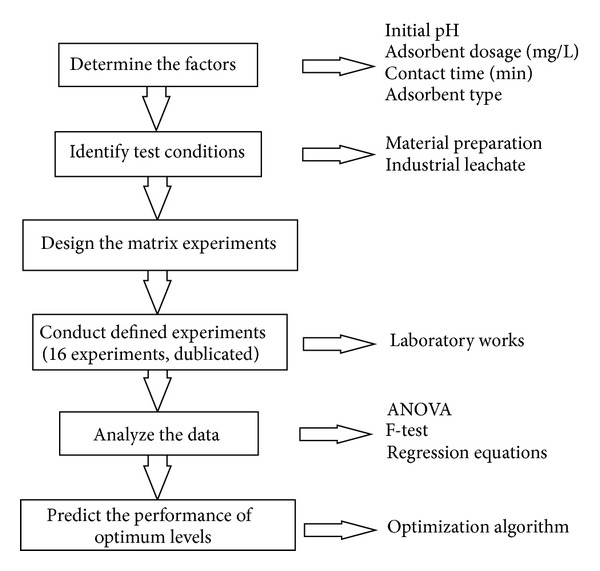
Cu(II) removal system based on statistical approach by montmorillonite.

In Figure 8, initial pH and adsorbent dosage are selected as main variables, while adsorbent type and contact time are selected as panel variables. Moreover, the quality characteristic of Cu(II) removal is measured at two extremes (initial pH and adsorbent dosage), and these measurements are plotted as vertical lines connecting the minimum and maximum values over the panel variables.  Figure 9 shows the general flowchart of the suggested batch experimental design.

### 2.6. Cascade Forward Neural Network (CFNN)

This paper presents an effective way to modeling batch adsorption system for Cu(II) removal. Nowadays, considerable achievements in artificial intelligence techniques can be used to model and predict the responses in complex systems. These techniques can enhance the predicting ability of the model such as adsorption systems if the mathematical or statistical methods fail to formulate with desired accuracy.

Many researchers present ANN techniques for modeling batch experimental systems. Generally, feed-forward backpropagation (FFBP) ANNs were successfully used in adsorption studies [42–48]. Details about ANNs can be found in the literature. All these techniques use more or less the same network architecture. The optimum network type is found by trial and error, and training procedures for these suggested ANNs need long computer runs.

FFBP consists of one input layer, one or several hidden layers, and one output layer. Backpropagation (BP) learning algorithm is usually used for learning procedure. The mathematical background of BP algorithm can be found in [49, 50].

The proposed modeling approach is different from the previous studies and is based on cascade forward neural network (CFNN) with 4 neurons in input layer, 8 neurons in hidden layer, and 1 neuron in output layer. The proposed CFNN is trained such that a particular input leads to a specific target output. CFNN is similar to FFBP network in using the BP algorithm for weights updating, but the main characteristic of this network is that neurons in each layer relate to neurons in all previous layer and avoid the drawbacks that occur with FFBP.

**Figure 10:**
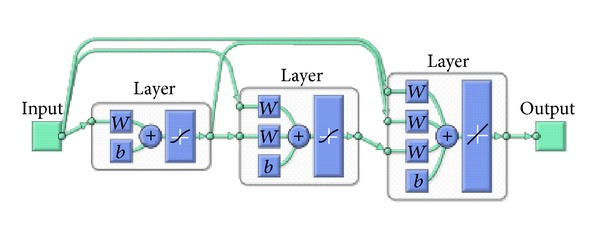
Proposed CFNN architecture for Cu(II) removal system.

The input and target data were selected from Table 2. The odd numbers of analyzed factors and removal efficiencies were used for training procedures, while the even ones were used for testing purposes. Training process consists of four steps: (a) assemble the training data, (b) decide the network type, (c) train the network, and (d) calculate the output for test data. Unlike the 2^4^ full factorial experimental design, the proposed CFNN uses only 8 inputs and responses out of 16. Therefore, it is easy to implement and cost-effective. The proposed network type is given in Figure 10.

**Figure 11:**
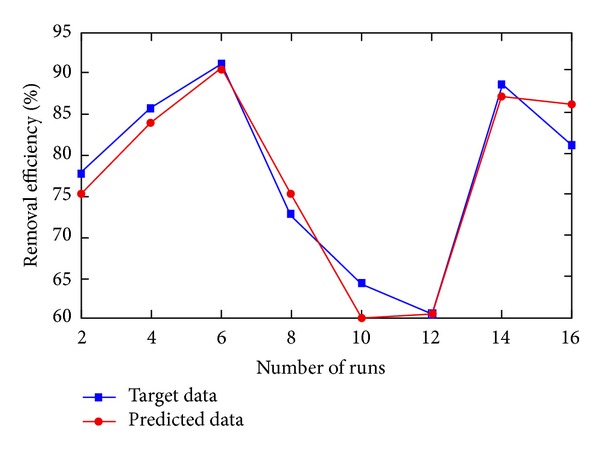
Testing procedure of the suggested CFNN.

Input and hidden layers consist of tangent sigmoid functions and output layer consists of linear function. The network type of 4-8-1 neurons in each layer was found to be optimum, with the lowest mean squared error and the highest determination of coefficient in training step. The actual and predicted experimental values for testing data are shown in Figure 11.

**Figure 12:**
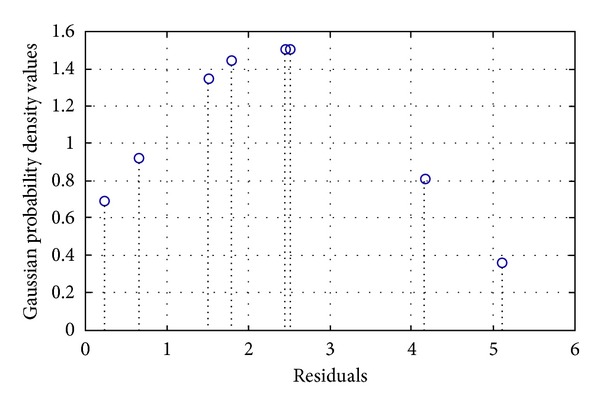
Gaussian probability density function of the residuals obtained from CFNN.

The determination of coefficient (*R* ^2^) was calculated as 0.9384 after 219 iterations. The network parameters such as weights and biases were updated according to the resilient backpropagation algorithm, which was found to be more effective than the other training algorithms.  Figure 12 shows the Gaussian probability density values of the residuals. It can be assumed that the errors are normally distributed and the CFNN model can be used for prediction purpose with reasonabe accuracy.

**Figure 13:**
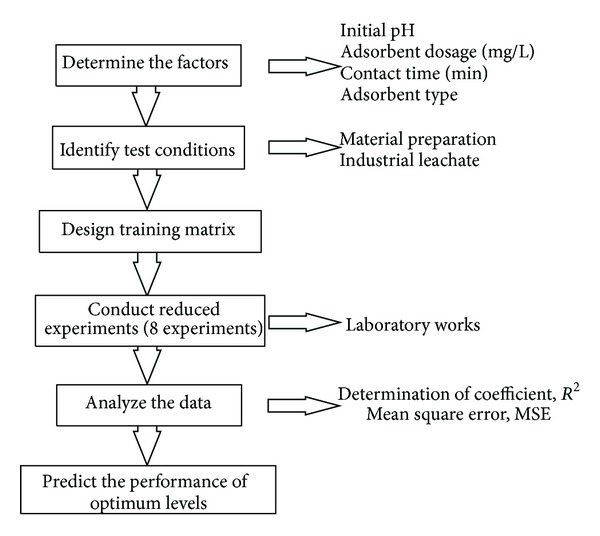
Cu(II) removal system based on CFNN by montmorillonite.

Figure 13 shows the proposed adsorption modeling system by using CFNN, which is easy to implement and presents an effective way to model the biosorption procedure by montmorillonite.

## 3. Conclusion

The idea of the study was to examine the feasibility of using montmorillonite as a possible adsorbent from industrial leachate for the removal of Cu(II). The following outcomes can be derived from this ongoing research work.

- FFED is definitely an acceptable method for studying the influence of all analyzed factors on response variable by considerably reducing the experimental runs.
- However, the proposed CFNN approach is easy to implement and is adopted to predict the response of the process. The model can be further extended to include more variables and experimental data to increase reliability.
- The optimum conditions for maximum removal of Cu (II) from industrial leachate of 80.7% and 88.91% depend on the adsorbent type.
- It can be concluded that montmorillonite can be regarded a low-cost alternative for removal of toxic metal ions from aqueous solutions.

## Figures and Tables

**Table 1:** Chemical compositions of the materials.

Components	w/w (%)	
	Montmorillonite	Industrial waste
Na_2_O	1.71	2.04
MgO	3.96	1.85
Al_2_O_3_	18.64	3.01
SiO_2_	64.11	23.18
CaO	2.37	11.21
K_2_O	0.50	0.37
Fe_2_O_3_	3.01	39.17
ZnO	—	14.15
CuO	—	0.71
SO_3_	—	7.09

**Table 2:** Experimental range and levels of independent variables.

Factors	Range and levels (actual)	
	Low	High
*A*	3	6
*B*	5	25
*C*	10	30
*D*	0	1

For *D*, 0 represents raw and 1 represents modified montmorillonite.

**Table 3:** 2^4^ full factorial experimental design with actual levels.

Runs	*A*	*B*	*C*	*D*	*R*% actual	*R*% predicted	Residuals
1	6	25	10	1	98.4	98.21	0.187
2	6	5	10	0	77.7	78.11	−0.412
3	3	25	10	0	69.7	70.00	−0.298
4	6	5	10	1	85.8	85.81	−0.012
5	3	5	30	1	71.2	71.81	−0.613
6	6	25	30	0	91.2	90.79	0.412
7	3	25	10	1	80.1	80.24	−0.138
8	3	25	30	0	72.7	73.06	−0.363
9	6	5	30	1	89.4	88.74	0.663
10	3	5	30	0	64.2	64.01	0.187
11	3	5	10	1	68.1	68.14	−0.038
12	3	5	10	0	60.4	59.94	0.462
13	3	25	30	1	83.7	82.91	0.787
14	6	25	10	0	88.7	88.46	0.237
15	6	25	30	1	99.3	100.14	−0.838
16	6	5	30	0	81.2	81.44	−0.238

*R*%: removal efficiency.

**Table 4:** Estimated effects and coefficients for *R*% (coded units).

Term	Effect	Coefficient	SE	*t*-test	*P* value	Remark
Constant		80.1125	0.1971	406.48	0.000	Significant
*A*	17.7000	8.8500	0.1971	44.90	0.000	Significant
*B*	10.7250	5.3625	0.1971	27.21	0.000	Significant
*C*	3.0000	1.5000	0.1971	7.61	0.001	Significant
*D*	8.7750	4.3875	0.1971	22.26	0.000	Significant
*A*∗*B*	0.1500	0.0750	0.1971	0.38	0.719	
*A*∗*C*	−0.3750	−0.1875	0.1971	−0.95	0.385	
*A*∗*D*	−0.2500	−0.1250	0.1971	−0.63	0.554	
*B*∗*C*	−0.5000	−0.2500	0.1971	−1.27	0.260	
*B*∗*D*	1.0250	0.5125	0.1971	2.60	0.048	Significant

SE: standard errors, standard deviation = 0.78, PRESS = 31.82, *R* ^2^ = 99.85%,*R* ^2^ (predicted) = 98.46%, and *R* ^2^ (adjusted) = 99.55%.

**Table 5:** Analysis of variance for *R*% (coded units).

Source	DF	Seq. SS	Adj. SS	Adj. MS	*F* value	*P* value
Main Effects	4	2057.26	2057.26	514.316	827.54	0.000
Two-Way interactions	6	6.26	6.26	1.044	1.68	0.293
Residual error	5	3.11	3.11	0.622		

Total	15	2066.64				

**Table 6:** Optimization of the analyzed factors.

Runs	Optimized factors	Predicted removal efficiency %
1	*A* = 5, *B* = 5, *C* = 10, *D* = 0 (1)	72.05 (79.92)
2	*A* = 5, *B* = 10, *C* = 10, *D* = 0 (1)	74.61 (82.99)
3	*A* = 6, *B* = 5, *C* = 10, *D* = 0 (1)	78.11 (85.81)
4	*A* = 6, *B* = 10, *C* = 10, *D* = 0 (1)	80.70 (88.91)
5	*A* = 6, *B* = 15, *C* = 10, *D* = 0 (1)	83.28 (92.01)
6	*A* = 6, *B* = 20, *C* = 10, *D* = 0 (1)	85.87 (95.11)
